# Autonomic Neuromodulation for Preventing and Treating Ventricular Arrhythmias

**DOI:** 10.3389/fphys.2019.00200

**Published:** 2019-03-11

**Authors:** Yanqiu Lai, Lilei Yu, Hong Jiang

**Affiliations:** ^1^ Department of Cardiology, Renmin Hospital of Wuhan University, Wuhan, China; ^2^ Cardiovascular Research Institute, Wuhan University, Wuhan, China; ^3^ Hubei Key Laboratory of Cardiology, Wuhan, China

**Keywords:** cardiac autonomic nervous system, neurocardiology, neuromodulation, neurorebalance, ventricular arrhythmias

## Abstract

The cardiac autonomic nervous system (CANS) is associated with modulation of cardiac electrophysiology and arrhythmogenesis. In this mini review, we will briefly introduce cardiac autonomic anatomy and autonomic activity in ventricular arrhythmias (VAs) and discuss novel approaches of CANS modulation for treating VAs. Studies over the decades have provided a better understanding of cardiac autonomic innervation and revealed overwhelming evidence of the relationship between autonomic tone and VAs. A high sympathetic tone and low parasympathetic (vagal) tone are considered as the major triggers of VAs in patients with myocardial ischemia, which can cause sudden cardiac death. In recent years, novel methods of autonomic neuromodulation have been investigated to prevent VAs, and they have been verified as being beneficial for malignant VAs in animal models and humans. The clinical outcome of autonomic neuromodulation depends on the level of cardiac neuraxis, stimulation parameters, and patient’s pathological status. Since autonomic modulation for VA treatment is still in the early stage of clinical application, more basic and clinical studies should be performed to clarify these mechanisms and optimize autonomic neuromodulation therapies for patients with VAs in the future.

## Introduction

**Figure 1:**
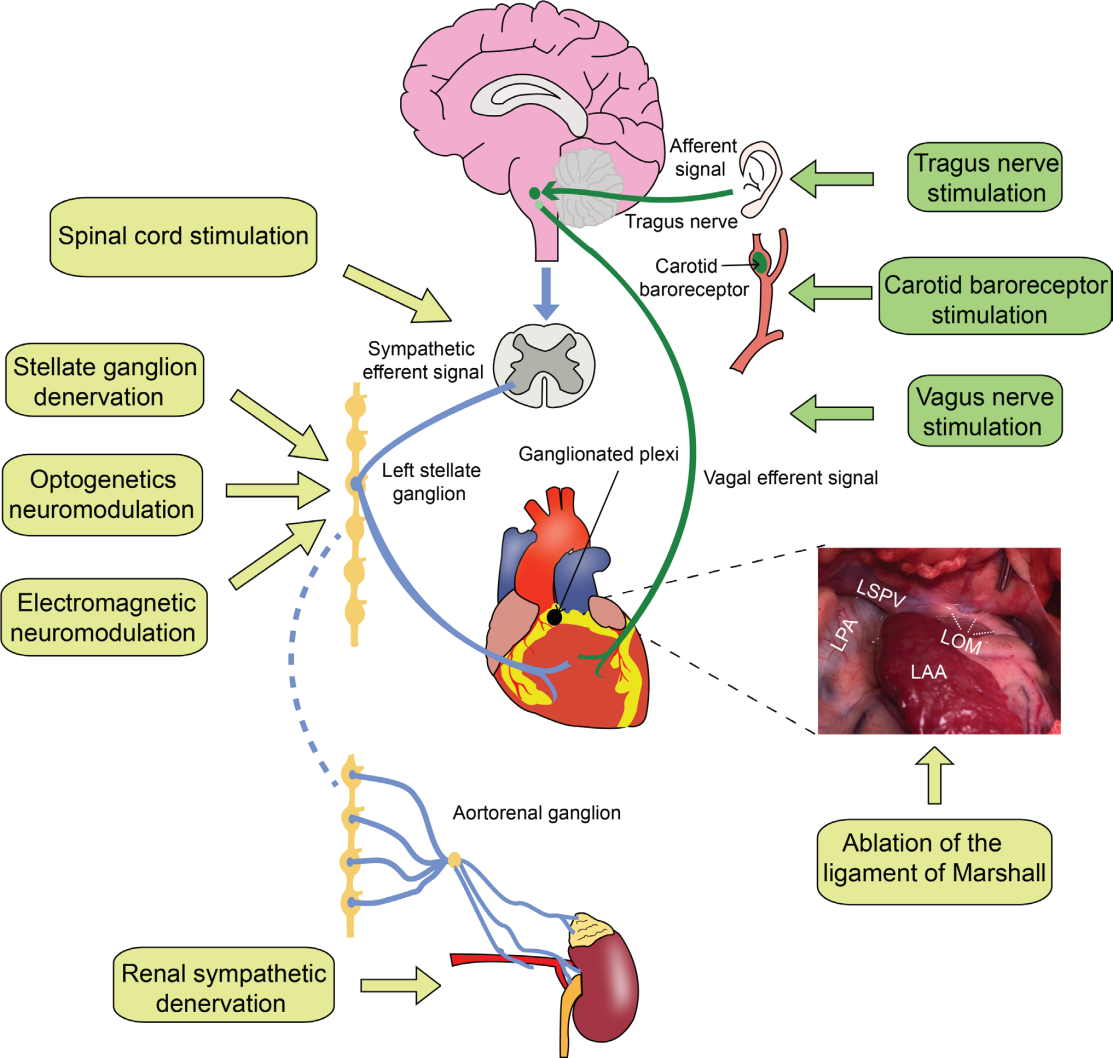
Cardiac autonomic innervation and neuromodulation strategies. LPA, left pulmonary artery; LSPV, left superior pulmonary vein; LOM, the ligament of Marshall; LAA, left atrial appendage. The blue lines represent sympathetic nerve fibers, and the green lines represent vagus nerve fibers.

The heart is an involuntary motor organ abundantly innervated by the autonomic nervous system. Although cardiac autonomic innervation has been studied in terms of anatomy or electrophysiological functions, the relationship between the autonomic nerves and different arrhythmogenesis is considered complex (Ellison and Williams, 1969; Fukuyama, 1982; Chen et al., 2007). Sympathetic hyperactivity or a low vagal tone contributes to the genesis of malignant VAs in myocardial infarction (MI), which can cause sudden cardiac death (Lombardi et al., 1983; Zhou et al., 2008; Priori et al., 2016). Neuromodulation of the cardiac autonomic nervous system (CANS) to rebalance the sympathetic and parasympathetic tones is very important for preventing VAs. Selectively modulating different components of the CANS is extremely beneficial for treating VAs. Herein, we will introduce cardiac autonomic anatomy and autonomic activity in VAs and discuss different strategies of autonomic intervention for treating VAs (Figure 1).

## Cardiac Autonomic Nerve Innervation

Decades of research on the anatomy of the CANS has improved understanding of the cardiac nerve innervation (Axford, 1928; Becker and Grunt, 1957; Ellison and Williams, 1969; Fukuyama, 1982; Kawashima, 2005). The CANS can be divided into extrinsic and intrinsic components. The extrinsic CANS contains sympathetic and parasympathetic parts mediating connections between the autonomic system and heart (Kawashima, 2005). The extrinsic sympathetic neurons are located in the brain, spinal cord, and paravertebral sympathetic ganglia. The sympathetic nerve fibers directly innervating heart are derived from these paravertebral ganglia. The extrinsic cardiac parasympathetic nerves are mainly derived from the medulla oblongata and routed *via* the vagus nerve, converging at the fat pads of the epicardium (Chiou et al., 1997). The intrinsic CANS contains autonomic neurons and nerves distributed on the surface of the heart (Armour et al., 1997). These neurons almost converge into the ganglionated plexi within the epicardial fat pads, which may act as an “integration center” in modulating the interactions between the extrinsic and intrinsic autonomic nerves (Armour et al., 1997; Pauza et al., 2002; Kawashima, 2005; Hou et al., 2007).

## Abnormal Autonomic Tone in VAs

Mechanisms of VAs are associated with the alteration of autonomic nervous activity, augmented sympathetic reflexes, or reduced vagal tone (Gilmour, 2001; Antzelevitch, 2007). In the ventricle, increased sympathetic tone, which may induce abnormal focal activity and reentrant activity, plays an important role in the genesis and maintenance of VAs (Lown and Verrier, 1976; Randall et al., 1976; Schwartz and Stone, 1980; Schwartz et al., 1985; Coumel, 1993). Shusterman et al. (1998) found that sympathetic activity reflexed by heart rate variability analysis was increased before the onset of ventricular tachyarrhythmias (VTs). Sympathetic hyperactivity can induce instability of the cardiac electrophysiological properties and facilitate the occurrence of VAs, especially in the ischemic heart (Lombardi et al., 1983; Inoue and Zipes, 1987; Charpentier et al., 1993; Bunch et al., 2007; Fukuda et al., 2015; Priori et al., 2016). Shortened ventricular effective refractory potential, action potential duration, fibrillation threshold, and triggered early and delayed after depolarization by high sympathetic tone increase the risk of VAs (Wang et al., 2017; Yu et al., 2017b). In addition to the changes of the cardiac electrophysiological properties due to hyperactivity of sympathetic nerves, structural remodeling of the sympathetic nerves occurs due to the presence of ischemic myocytes. The density of nervous synapses and nerves of the left stellate ganglion (LSG) is increased in dogs with acute MI (Cao et al., 2000; Han et al., 2012). Moreover, c-fos, the marker of sympathetic activity, was also upregulated in LSG and at the infarcted site (Wang et al., 2017). The remote effect of this remodeling further causes the cardiac electrophysiological properties more unstable. Patients with a history of VT and ventricular fibrillation (VT/VF) had increasing sympathetic nerve sprouting (Cao et al., 2000). This histological evidence indicates that the cardiac pathological state induced by ischemia can influence sympathetic nerve activity and further exacerbate the occurrence of VAs, forming a vicious cycle between the heart and sympathetic nerve system.

## Methods of Autonomic Modulation for Treating Ventricular Arrhythmias

### Carotid Baroreceptor Stimulation

The carotid sinus, an area of expansion at the beginning of the internal carotid artery, contains numerous baroreceptors functioning as a modulator for maintaining blood pressure. The baroreceptor component of the carotid sinus nerve originating from glossopharyngeal nerves is located in the thickened adventitia of the carotid sinus and elicits sympatho-inhibition (Porzionato et al., 2018). VAs have been associated with depressed baroreflex sensitivity (Schwartz et al., 1988; La Rovere et al., 2002). In conscious dogs with healed MI, reduction in the MI-reduced baroreflex sensitivity was associated with a higher risk of ventricular fibrillation (Schwartz et al., 1988). However, an exercise-induced increase in baroreflex sensitivity serving as an autonomic marker of increased vagal activity can reduce post-MI mortality (La Rovere et al., 2002). Our study demonstrated that electrical carotid baroreceptor stimulation (CBS) has therapeutic potential for VAs by stabilizing the ventricular electrophysiological properties in dogs with MI (Liao et al., 2014a). Since high-level CBS may induce atrial arrhythmias by activating vagal nerve, low-level CBS has been investigated in the ischemic heart for preventing VAs. Our group found that low-level CBS with 80% intensity of the threshold needed to slow the heart rate could prolong the ventricular effective refractory potential and significantly reduce the incidence of VT/VF during acute MI (Liao et al., 2014b; Zhou et al., 2015). The inhibition of LSG neural activity might be involved in the underlying mechanism of low-level CBS. The observations in animals provide the proof of concept that CBS can improve imbalance between sympathetic and parasympathetic nerve activity. CBS is invasive and the fact that the carotid sinus is located in such a complex area may impede the development of CBS for treating VAs in the clinical setting.

### Spinal Cord Stimulation

Spinal cord stimulation (SCS) has been globally used to treat peripheral vascular disease, Raynaud disease, refractory angina, and neuropathic pain through implantation of a stimulating electrode in the epidural space of the spinal canal (Ekre et al., 2002). The ability of SCS to modulate cardiac sympathetic innervation in the treatment of ischemic VAs has been investigated recently. Animal models demonstrated that SCS could decrease the occurrence of ischemia-induced VAs (Issa et al., 2005; Lopshire et al., 2009; Wang et al., 2015; Howard-Quijano et al., 2017). For example, SCS (50 Hz, 0.2-ms pulse width) applied at the T_1_-T_5_ spinal cord level for 1 h reduced acute MI-induced VAs by suppressing LSG activity in dogs with MI (Wang et al., 2015). Additionally, in dogs with heart failure, Lopshire and Zipes (2014) revealed that neuromodulation with SCS improved left ventricular function and decreased the incidence of VT. In patients with a high risk of VT/VF, chronic SCS could help decrease the incidence of VAs through stabilization of the cardiac electrophysiological properties (Ferrero et al., 2008; Grimaldi et al., 2012). Grimaldi et al. demonstrated that two patients with cardiomyopathy who experienced a sustained VT episode or an episode of ventricular fibrillation received long-term SCS at the T_3_ level, and both types of VT episodes were reduced (Ferrero et al., 2008). A prospective, multicenter, first-in-human trial (SCS HEART study) that studied the efficacy of SCS in patients with systolic heart failure showed that high thoracic (T_1–3_) SCS (50 Hz, 200-ms pulse width) potentially improves symptoms, the cardiac functional status, and left ventricular function and remodeling (Tse et al., 2015). Decreased sympathetic tone and enhanced vagal tone might underlie the antiarrhythmic effects of SCS. It is necessary to perform more studies on the long-term effects and safety of SCS since the potential mechanisms are still unclear.

### Stellate Ganglion Modulation

#### Denervation

LSG hyperactivity can precede most malignant VAs (Schwartz et al., 2004; Zhou et al., 2008). Modification of cardiac sympathetic nerve activity through stellate ganglion ablation has been extensively studied and its effects on reducing VAs has been revealed (Vaseghi et al., 2014). Left cardiac sympathetic denervation or stellate ganglion blockade with β-blockers have significantly reduced the incidence of recurrent VAs and increased survival (Steinbeck et al., 1992; Nademanee et al., 2000; Coleman et al., 2012; Schneider et al., 2013). Jonnesco (1921) first reported left cardiac sympathetic denervation in one patient with incapacitating angina accompanied with life-threatening VAs, and it successfully stopped angina and VAs (Schwartz et al., 2017). Subsequently, left cardiac sympathetic denervation has been explored for treating drug-resistant VT and inherited arrhythmia syndromes because it exhibited a protective effect against cardiac death in high-risk patients after MI, thereby making it an alternative to β-blockers (Schwartz et al., 1992, 2004; Napolitano and Priori, 2007; Vaseghi et al., 2017b). Moreover, bilateral stellate ganglion denervation has been successfully used to prevent VT/VF storms heart failure, and it is more effective than left cardiac sympathetic denervation in preventing VAs (Vaseghi et al., 2014). However, there may be some side effects associated with stellate ganglion ablation surgery such as Horner’s syndrome, unexpected hemorrhage, unilateral hand dryness, abnormal sweating, chest pain, and incomplete denervation that impede its clinical use (Schwartz et al., 2004; Bourke et al., 2010; Odero et al., 2010).

#### Electromagnetic Fields

Electromagnetic fields (EMF) have been used as a therapy in autonomic disorders (Anninos et al., 1991; Sandyk, 1992a,b). EMF can significantly change the physiological properties of the CANS neural network by causing some ionic flux (Mathie et al., 2003; Saunders, 2003; Bistolfi, 2007). Wang et al. (2016b) showed that low-frequency EMF (1 Hz, intensity at approximately 90% of the motor threshold; 8 s on, 10 s off) stimulated the surface area at which the LSG is located and reduced the neural activity of the LSG and the occurrence of MI-induced VAs in canines. LSG neuromodulation by EMF may become a novel approach for treating heart diseases associated with autonomic nerve dysfunction. The effects of EMF on arrhythmogenesis can be altered by using EMF with different frequencies and amplitudes. Although few clinical studies have been performed to investigate the anti-arrhythmic effect of EMF for patients with VAs, EMF may be a promising noninvasive approach for treating VAs.

#### Optogenetics

Optogenetics combines optical stimulation and genetic modification of target cells. It has been widely used in selectively stimulating specific neurons in neural tissues (Boyden et al., 2005; Zhang et al., 2007), thereby resulting in silencing or enhancement of neural activity (Deisseroth, 2011; Yizhar et al., 2011). Our team first used optogenetics in modulating LSG neurons to prevent VAs in canines with acute MI (Yu et al., 2017c). ArchT proteins were expressed in the LSG neurons through virus transfection. ArchT is an inhibitory light-sensitive opsin that can be activated by proper illumination (565 nm), leading to hyperpolarizing currents in the neurons. Our study demonstrated that the activity of gene-modified LSG neurons could be reversely decreased by light, and suppressed LSG exerted a protective effect against post-infarcted VAs. The underlying mechanisms may include induced neural hyperpolarization and reduction of c-fos and nerve growth factor levels in the LSG neurons. This proof-of-concept study provided the possibility of using optogenetics for modulating cardiac neurons to prevent ischemia-induced VAs. Since researchers have developed implantable wireless optogenetic devices, remote LSG neuromodulation *via* subcutaneous implantation of a wireless light transmitter may be possible in the future (Montgomery et al., 2015; Park et al., 2015). Further large-scale studies should be continuously performed to determine its long-term effect and safety.

### Selective Ablation of the Ligament of Marshall

The ligament of Marshall (LOM) contains nerves, the vein, and muscle tracts, and it is a remnant of the left superior vena cava (Scherlag et al., 1972). The proximal portion of the LOM connecting to the coronary sinus is primarily innervated by parasympathetic nerves, while the distal portion extending to the left superior pulmonary vein (LOM_LSPV_) is mainly innervated by sympathetic nerves (Kim et al., 2000; Makino et al., 2006). LOM ablation has been extensively investigated to treat atrial arrhythmias, but few studies focused on the relationship between the sympathetic elements in the LOM and VAs (Hwang et al., 2000, 2006). In canines, Lin et al. (2008) found that neural activation of the LOM_LSPV_ by norepinephrine injection caused accelerated junctional rhythms, idioventricular rhythms, and VT. LOM_LSPV_ ablation reduced VAs occurrence induced by cesium combined with LSG stimulation in canines (Wang et al., 2016a). Additionally, in dogs with acute MI or ischemia-reperfusion models, LOM_LSPV_ ablation ameliorated the prevalence of VAs by reducing sympathetic tone (Liu et al., 2018; Ma et al., 2018). Therefore, the LOM may serve as a sympathetic conduit between the LSG and ventricles. The antiarrhythmic effects of LOM ablation might be due to cellular damage and tyrosine hydroxylase-negative and terminal deoxynucleotidyl transferase-mediated dUTP-biotin nick end labeling-positive cells increasing in the LSG, which reduces sympathetic communication between the extrinsic and intrinsic nerves of the heart (Zhao et al., 2016).

### Vagal Nerve Stimulation

#### Cervical Vagal Nerve Stimulation

Cervical vagal nerve stimulation (VNS) has demonstrated antagonizing proarrhythmic sympathetic tone during MI. Multiple studies have shown promising results that augmented vagal tone increases ventricular electrical stability and protects against VAs during acute ischemia in animal models (Goldstein et al., 1973; Corr and Gillis, 1974; Myers et al., 1974; Takahashi et al., 1998). In dogs with healed MI, VNS (3–8 Hz, 3 ms, 1.0–3.0 mA) that reduced the heart rate after the onset of acute ischemic episodes significantly prevented ventricular fibrillation from 100 to 10% (Vanoli et al., 1991). Furthermore, VNS was found to stabilize the infarct-border zone and reduce the occurrence of VT in a chronic porcine infarct model (Vaseghi et al., 2017a). Additionally, low-amplitude VNS (20 Hz, 500-μs pulse width, 3.5 mA) using a continuous or intermittent mode that slightly alters the PR interval could prevent myocardial ischemia/reperfusion (R/I) injury in swine (Shinlapawittayatorn et al., 2013). Our study also found that low-level VNS (20 Hz, 0.1-ms duration) with a stimulation voltage below the 80% voltage threshold required to slow the heart rate significantly reduced myocardial R/I injury in canines (Chen et al., 2016). Although no clinical study has investigated the efficacy of VNS for treating VAs in patients, clinical trials have been used to evaluate the effect of VNS on cardiac function of patients with heart failure (Premchand et al., 2014, 2016; Zannad et al., 2015). The beneficial effects of VNS on treating VAs mainly involve mechanisms such as slowing the heart rate, reducing sympathetic activity, and inhibiting renin angiotensin aldosterone system activation (Vanoli et al., 1991). Another important antiarrhythmic mechanism of VNS may be related to histological remodeling of the LSG, which decreases the sympathetic tone and activation of the classical cholinergic anti-inflammatory pathway (Inoue et al., 2016). Although cervical VNS can exert an antiarrhythmic effect in ischemic hearts, it may increase the risk of adverse effects such as infection, dysphagia, hoarseness, cough, and pain due to the surgical implantation of a neurostimulator around the cervical vagal nerve (Spuck et al., 2010; Elliott et al., 2011; Premchand et al., 2014; Zannad et al., 2015).

#### Transcutaneous Vagus Stimulation

The auricular branch of the vagal nerve is the only nerve branch located on the body surface that can conduct peripheral information to the brain. Tragus nerve stimulation has been investigated as a noninvasive alternative to VNS. In conscious dogs with healed MI, chronic low-level tragus stimulation (LL-TS) attenuated left ventricular remodeling, and reduced cardiac autonomic remodeling and VAs inducibility probably by decreasing activity of the LSG in a post-infarcted canine model (Wang et al., 2014; Yu et al., 2016). Our group provided the first clinical evidence that the LL-TS (20 Hz, 1-ms duration; 5 s on, 5 s off) at 50% below the threshold reduces myocardial ischemia reperfusion injury and ischemia-reperfusion-associated VAs, and improves cardiac function in patients with ST-segment elevation MI undergoing primary percutaneous coronary intervention (Yu et al., 2017a). LL-TS is a potential noninvasive intervention for autonomic modulation in patients with prevalent VAs. However, further research on the mechanisms and larger scale clinical trials are needed to understand the long-term efficacy and safety of LL-TS.

### Renal Sympathetic Denervation

Renal sympathetic nerves, either afferent or efferent components, play an important role in modulating central sympathetic activity. Although renal sympathetic denervation (RSD) has been extensively investigated to decrease blood pressure in animals and patients with refractory hypertension, it appears to influence autonomic activity, which is an effect beyond blood pressure reduction (Krum et al., 2009; Symplicity et al., 2010; Linz et al., 2013a,b; van Brussel et al., 2015; Jackson et al., 2017). In an ischemic canine model, renal nerve stimulation increased LSG neural activity, thus facilitating ischemia-induced VAs (Huang et al., 2014). Renal sympathetic ablation decreased the occurrence of VAs probably by inhibiting cardiac sympathetic activity in a cesium-induced long QT canine model (Yu et al., 2017b). Furthermore, RSD reduced the numbers of spontaneous VAs in pigs with ischemia or MI (Linz et al., 2013b; Jackson et al., 2017). Ukena et al. (2012) first reported that RSD reduced ventricular arrhythmic episodes successfully in two patients with chronic heart failure suffering from drug-resistant electrical storm and decreased VT episodes in three patients with cardiomyopathy and recurrent refractory VT as an adjunctive therapy (Remo et al., 2014). These studies provide evidence of a potential link between renal sympathetic nerves and cardiac sympathetic nerves (kidney-heart cross talk). However, RSD could significantly compromise the compensatory hemodynamic response to hypotensive hemorrhage (Singh et al., 2017), thereby highlighting the potential challenge for the clinical application of RSD. Therefore, future investigations about the long-term effects and safety of RSD are needed.

## Conclusions

Autonomic dysfunction is a significant hallmark of heart disease. Imbalance between the sympathetic and parasympathetic limbs of the CANS is involved in the genesis of VAs and a contributor to VA progression. Different approaches to modulating autonomic dysfunction in VAs focus on reducing sympathetic hyperactivity or enhancing the vagal activity, and have aroused increasing interest recently. The human heart is innervated by such an intricate neural network; however, a better understanding of the CANS is still necessary to treat VAs precisely and effectively. Although there is no direct evidence of relationship between central nervous system and cardiac arrhythmia, it should not be ignored that central nervous system also plays a dominant role in the autonomic modulation on cardiac arrhythmia when talking about these modulation strategies. The outcome of autonomic neuromodulation depends on the level of cardiac neuraxis, stimulation parameters and patient’s pathological status. Since some autonomic modulation strategies for VAs only stay at the animal stage due to their invasiveness and side-effects, autonomic modulation for VA treatment is still in the early stage of clinical application. More basic and clinical studies should be performed to develop safer and more effective ones as well as clarifying their mechanisms and optimizing the stimulation parameters for patients with VAs in the future.

## Author Contributions

All authors listed have made a substantial, direct and intellectual contribution to the work, and approved it for publication.

### Conflict of Interest Statement

The authors declare that the research was conducted in the absence of any commercial or financial relationships that could be construed as a potential conflict of interest.

## Abbreviations

CANS: Cardiac autonomic nervous system
CBS: Carotid baroreceptor stimulation
EMF: Electromagnetic fields
LL-TS: Low-level tragus stimulation
LOM: The ligament of Marshall
LSG: Left stellate ganglion
MI: Myocardial infarction
RSD: Renal sympathetic denervation
SCS: Spinal cord stimulation
VAs: Ventricular arrhythmias
VNS: Vagal nerve stimulation
VT: Ventricular tachyarrhythmias
VT/VF: Ventricular tachycardia and fibrillation.
